# Innovative Applications of Nanopore Technology in Tumor Screening: An Exosome-Centric Approach

**DOI:** 10.3390/bios15040199

**Published:** 2025-03-21

**Authors:** Heng Chi, Liuxin Shi, Songlin Gan, Guangyi Fan, Yuliang Dong

**Affiliations:** 1 BGI Research, Shenzhen 518083, China; chiheng@genomics.cn (H.C.); shiliuxin@genomics.cn (L.S.); 2 BGI Research, Hangzhou 310030, China; gansonglin@genomics.cn; 3 BGI Research, Qingdao 266555, China

**Keywords:** nanopore, exosome, tumor screening, medical devices

## Abstract

Cancer remains one of the leading causes of death worldwide. Its complex pathogenesis and metastasis pose significant challenges for early diagnosis, underscoring the urgent need for innovative and non-invasive tumor screening methods. Exosomes, small extracellular vesicles that reflect the physiological and pathological states of their parent cells, are uniquely suited for cancer liquid biopsy due to their molecular cargo, including RNA, DNA, and proteins. However, traditional methods for exosome isolation and detection are often limited by inadequate sensitivity, specificity, and efficiency. Nanopore technology, characterized by high sensitivity and single-molecule resolution, offers powerful tools for exosome analysis. This review highlights its diverse applications in tumor screening, such as magnetic nanopores for high-throughput sorting, electrochemical sensing for real-time detection, nanomaterial-based assemblies for efficient capture, and plasmon resonance for ultrasensitive analysis. These advancements have enabled precise exosome detection and demonstrated promising potential in the early diagnosis of breast, pancreatic, and prostate cancers, while also supporting personalized treatment strategies. Additionally, this review summarizes commercialized products for exosome-based cancer diagnostics and examines the technical and translational challenges in clinical applications. Finally, it discusses the future prospects of nanopore technology in advancing liquid biopsy toward clinical implementation. The continued progress of nanopore technology not only accelerates exosome-based precision medicine but also represents a significant step forward in next-generation liquid biopsy and tumor screening.

## 1. Introduction

Cancer is one of the leading causes of global mortality, driven by its complex pathogenesis and high propensity for metastasis [1,2,3,4]. Metastatic spread often remains undetected until advanced stages [5,6,7,8,9], severely complicating treatment and underscoring the urgent need for early detection [10,11,12,13,14,15]. Traditional tissue biopsy methods, though widely used, are invasive and limited in their ability to capture tumor heterogeneity, often increasing the risk of metastasis. In contrast, liquid biopsy has emerged as a non-invasive, cost-effective alternative, offering dynamic insights into tumor biology through the analysis of circulating biomarkers [16,17,18,19,20].

Among liquid biopsy targets, exosomes are small extracellular vesicles (EVs) (30–150 nm) secreted by cells and they have gained prominence as molecular reservoirs that reflect the physiological and pathological states of their parent cells. These vesicles carry nucleic acids, proteins, and lipids that mediate intercellular communication, promote angiogenesis, and modulate immune responses within the tumor microenvironment [9,21,22,23,24,25,26,27,28]. Exosomal biomarkers, such as miRNAs, lncRNAs, and tumor-associated proteins, hold great promise for cancer detection, offering high specificity even at early disease stages. Specific miRNAs in circulating exosomes, for example, have shown high sensitivity and specificity for detecting cancers such as breast, lung, and liver cancer [29,30,31,32]. However, several challenges persist in translating their potential into clinical practice, including exosome heterogeneity, low abundance in biofluids, and limitations of current isolation and detection methods, such as ultracentrifugation and immunocapture, which often suffer from issues with purity, throughput, and reproducibility [33,34]. Nanopore technology has emerged as a promising solution to address these challenges. By leveraging nanoscale pores to detect electrical or optical perturbations caused by exosomes, nanopore technology enables single-particle resolution, real-time monitoring, and label-free detection [35,36,37,38]. Innovations, including magnetic nanopores for high-throughput sorting, electrochemical sensors for redox profiling, and plasmonic resonance arrays for ultrasensitive protein detection, have significantly enhanced exosome isolation and analysis [39,40,41,42,43,44,45,46]. These advancements improve sensitivity, specificity, and scalability, providing a solid foundation for clinical applications.

In this review, we aim to evaluate the role of exosomes in cancer diagnostics and the advancements in nanopore technology for exosome isolation and detection. We discuss the challenges in current exosome-based cancer diagnostics, including the heterogeneity of exosomes, issues with isolation methods, and the need for more efficient detection platforms. By focusing on the promising applications of nanopore biosensor technology, we explore how high-throughput separation, electrochemical sensing, and plasmon resonance techniques can improve the sensitivity, specificity, and efficiency of exosome analysis. Additionally, we provide case studies on nanopore-based exosome detection in tumor screening, focusing on their potential in early cancer detection and personalized treatment strategies. Through this review, we aim to highlight how nanopore technology could address existing limitations in exosome-based diagnostics and discuss its future clinical applications.

## 2. Exosomes and Tumor Screening

### 2.1. The Importance of Exosomes in Tumor Screening

Exosomes are EVs ranging from 30 to 150 nm in diameter, widely distributed in body fluids such as blood, urine, cerebrospinal fluid, and saliva [9,47,48,49]. Their biogenesis begins with the budding of the plasma membrane, forming multivesicular bodies (MVBs) that encapsulate intraluminal vesicles (ILVs). Upon fusion of MVBs with the plasma membrane, ILVs are released as exosomes [9,50]. Exosomes carry a variety of biomolecules, including proteins, RNA, and DNA, whose composition and heterogeneity reflect the phenotype, state, and functional activity of their parent cells [51,52,53]. According to the ExoCarta database, exosomes are known to contain up to 41,860 proteins, 7540 RNAs, and 1116 lipid molecules [54]. Exosomes exhibit significant heterogeneity, with even the same cell secreting exosomes of distinct compositions. The RNA and protein profiles of exosomes change in response to the physiological or pathological states of their parent cells or tissues [55]. Nucleic acids in exosomes include microRNAs (miRNAs), messenger RNAs (mRNAs), transfer RNAs (tRNAs), long non-coding RNAs (lncRNAs), and viral RNAs [52,56,57,58]. Proteins enriched in exosomes are primarily involved in membrane transport and fusion processes, such as tetraspanins (CD9, CD63, and CD81), heat shock proteins (HSP60 and HSP70), and ESCRT-related components (Alix and TSG101). These components are essential markers for identifying donor cells [9].

An increasing body of evidence demonstrates that the secretion of exosomes by cancer cells is significantly elevated. Studies indicate that about 200 trillion exosomes are present in the blood of healthy individuals, with this number increasing to 400 trillion in cancer patients, reflecting the unique physiological characteristics of cancer cells [59,60,61]. These exosomes not only transport molecules that contribute to tumor growth and metastasis but also influence the microenvironment of distant organs through long-range signaling, accelerating cancer progression [62,63]. Due to their presence in various bodily fluids, exosomes have become promising tools for liquid biopsy, offering significant potential for the early detection of cancers such as prostate, breast, pancreatic, glioblastoma, and melanoma [48,64,65,66]. Tumor-specific molecules found in exosomes have been identified as key diagnostic indicators in the early stages of cancer, particularly before metastasis occurs, providing a critical window for timely intervention [67,68]. Furthermore, the diverse molecular information carried by exosomes enables precise differentiation between cancer types and their distinct molecular profiles, facilitating the development of personalized therapeutic strategies [69]. In-depth analysis of the protein and nucleic acid composition of exosomes will offer valuable insights into their role in cancer detection and treatment, providing a solid foundation for their widespread adoption in the medical field [70].

### 2.2. The Advantages of Exosomes in Tumor Screening

Cancer, one of the leading global causes of death, presents significant challenges due to its complex progression and difficulty in early detection. Traditional methods like tissue biopsy, while commonly used, may cause discomfort and carry surgical risks [71,72]. More critically, their limited sensitivity often leads to early-stage cancers being missed, with detection occurring only at later stages [17,18,19]. To address these challenges, researchers are increasingly turning to non-invasive, highly sensitive diagnostic methods like liquid biopsy, which offer a promising and cost-effective alternative [20].

Among the three main branches of liquid biopsy—circulating tumor cells (CTCs), circulating tumor DNA (ctDNA), and exosomes—exosomes exhibit more pronounced clinical advantages. Compared to the relatively low abundance of CTCs in the bloodstream, exosomes are present at much higher concentrations (~10⁹ particles/mL), making them more accessible [28,73,74]. Their higher abundance also means that smaller sample volumes are sufficient for detection, minimizing the risk of reduced sensitivity due to inadequate sampling [75,76]. Exosomes can detect cancer biomarkers at extremely low concentrations, even before clinical symptoms appear, which is crucial for improving early treatment success rates and enhancing patient outcomes [77,78,79]. Additionally, certain exosomal subtypes can effectively identify the molecular signatures of cancer cells, offering potential for early intervention [70,77]. For example, the glycoprotein Glypican-1 (GPC1) on serum-derived exosomes from pancreatic cancer patients shows strong signals, making it a promising tool for early pancreatic cancer detection [59]. The high sensitivity and specificity of exosome-based detection highlight its significant advantages in cancer diagnosis and prognostic monitoring. Furthermore, the bilayer lipid membrane structure of exosomes provides exceptional stability, protecting them from enzymatic degradation in circulation. In contrast, secreted proteins and ctDNA in plasma are rapidly degraded, limiting their reliability. This stability allows exosomes to persist in the bloodstream for extended periods, making them reliable biomarkers for clinical diagnosis and monitoring [68,75,76].

Exosomes are abundantly present in various biofluids, including blood, urine, and saliva, allowing for isolation without invasive tissue sampling. This non-invasive method enhances patient comfort and acceptance, encouraging wider participation in regular screenings, which is essential for early cancer detection and prevention [80,81,82,83]. Additionally, the non-invasive nature of exosome-based detection makes it ideal for long-term monitoring of cancer progression. Clinicians can gain real-time insights into tumor development, its spread, and metastatic trends with high accuracy. Exosomes provide comprehensive information for cancer diagnosis. Unlike circulating tumor DNA (ctDNA), which is released from apoptotic or necrotic cells, exosomes are secreted by living cells and carry rich biomolecular information, including proteins, RNA, and DNA, derived from their parent cells. This allows exosomes to provide a more accurate representation of the physiological state and molecular characteristics of cancer cells [25,28]. The molecular biomarkers within exosomes form a distinct “molecular fingerprint” that is invaluable for molecular classification and subtyping of cancers, as well as for providing critical data to guide personalized therapeutic strategies [84].

Exosome-based detection has shown remarkable potential in the early diagnosis of cancer, owing to its high abundance, rich molecular information, remarkable stability, high sensitivity and specificity, as well as its non-invasiveness. With continuous advancements in exosome research and optimization of detection technologies, exosomes are set to become indispensable tools for early cancer detection and personalized therapy. Future diagnostic strategies leveraging exosomes are expected to enable earlier and more accurate cancer detection, ultimately improving patient prognosis and quality of life.

### 2.3. Applications of Multiple Biomarkers in Exosomes for Cancer Screening

Exosomes show great potential in liquid biopsy for cancer. Studies have demonstrated that exosomes can stably carry miRNA, lncRNA, circular RNA, and proteins, making them ideal for liquid biopsy [29,30].

Exosomal miRNAs have emerged as promising biomarkers across various cancers. In hepatocellular carcinoma (HCC), for instance, *miR-21* is significantly upregulated, promoting HCC cell proliferation, migration, and chemoresistance by suppressing tumor suppressor genes like *PTEN* and *PDCD4*. This makes *miR-21* a key biomarker for HCC diagnosis and prognosis [85]. Furthermore, Nakano et al. reported that exosomal *miR-92b* demonstrated superior diagnostic performance compared to the traditional biomarker alpha-fetoprotein (AFP) in HCC patients undergoing liver transplantation [86]. In pancreatic cancer (PDAC), the upregulation of exosomal *miR-10b* and its expression differences between PDAC, high-risk chronic pancreatitis, and healthy individuals highlight its potential as an early diagnostic biomarker [87,88,89]. Similarly, in breast cancer, exosomal miRNAs such as *miR-21*, *miR-222*, and *miR-200c* can complement traditional biomarkers like Cancer Antigen 15-3 (CA15-3), improving diagnostic accuracy by addressing their limited sensitivity [90]. In non-small cell lung cancer (NSCLC), exosomal miRNAs such as *miR-21*, *miR-378*, *miR-139*, and *miR-200* exhibit significantly altered expression compared to healthy individuals, expanding the biomarker repertoire for early NSCLC diagnosis [91]. Notably, urinary exosomes contain higher RNA levels than those from plasma or bronchoalveolar lavage fluid, emphasizing urine as a promising and non-invasive source for NSCLC biomarker discovery [92].

The application of exosomal lncRNAs, circRNAs and mRNAs in cancer detection is rapidly advancing [30,93,94]. For example, elevated serum levels of lncRNA *PCAT-14* in HCC patients show significant diagnostic value, correlating with disease progression in HCC [95,96]. Studies show that plasma levels of *hsa-circ-0013958* in lung cancer patients are positively correlated with tumor metastasis, making it a promising diagnostic and prognostic biomarker. Additionally, exosomal *TUBB3* mRNA is highly expressed in castration-resistant prostate cancer (CRPC) patients and strongly associated with shorter progression-free survival, further emphasizing its diagnostic and prognostic significance in prostate cancer [97].

Exosomes, which carry nucleic acids and various proteins, play a pivotal role in cancer diagnostics and monitoring by reflecting the biological status of tumor cells. In the early detection of PDAC, circulating exosomal GPC-1+ has demonstrated exceptional diagnostic accuracy, outperforming the traditional biomarker Carbohydrate Antigen 19-9 (CA19-9) [87]. Similarly, breast cancer (BC) diagnosis has benefited from research on exosomal proteins, including heat shock protein 70 (HSP70), thrombospondin-1 (TSP1), lactate dehydrogenase C4 (LDH-C4), exo-Anx2, and integrin α6, all of which show diagnostic potential and contribute to disease monitoring [98]. For prostate cancer (PCa), traditional detection relies on prostate-specific antigen (PSA); however, benign prostatic hyperplasia (BPH) and other inflammations often lead to elevated PSA levels, reducing its diagnostic accuracy [99]. In contrast, exosomal EphrinA2 shows superior performance in distinguishing prostate cancer from benign prostatic hyperplasia, significantly outperforming PSA-based detection [100].

Exosomes’ ability to carry multiple biomarkers in liquid biopsy significantly enhances their potential for cancer detection and monitoring. These biomarkers broaden the scope for identifying novel, specific biomarkers.

### 2.4. The Limitations of Exosomes in Tumor Screening

Although exosomes hold significant promise in cancer diagnosis due to the specific molecular information they carry, their practical application still faces substantial challenges [101,102,103,104,105,106].

The inherent heterogeneity of exosomes complicates their use as reliable cancer biomarkers. Variations in size, source cell types, and physiological states contribute to the diversity of exosomal components, making biomarker identification challenging [107]. Moreover, since exosomes are secreted by nearly all somatic cells, identifying cancer-specific biomarkers remains difficult. Even after potential biomarkers are identified through omics technologies, they require rigorous clinical validation and biological function analysis, a complex and time-consuming process that delays their clinical application [108]. Additionally, existing detection technologies often lack the sensitivity to capture low-abundance exosomal molecules, leading to false positives or missed detections [109]. Accurately distinguishing the origin of exosomes and understanding the molecular characteristics of different exosome types are also unresolved challenges, further limiting their potential in cancer detection.

From a technical perspective, various methods for exosome isolation and detection—such as differential centrifugation, ultracentrifugation, immunoaffinity capture, and size-based separation—have been developed. However, these techniques still face significant limitations in terms of purity, throughput, and specificity [33]. The small size and low density of exosomes, especially in complex biological fluids, make it challenging to eliminate contamination from other EVs and nonspecific proteins, compromising detection accuracy and reproducibility [33,34]. These impurities not only reduce exosome purity but also interfere with subsequent analyses, undermining reliability further. Moreover, the lack of standardized protocols for isolation, characterization, and analysis exacerbates inconsistencies across studies, limiting comparability and reproducibility and hindering clinical translation [110]. Addressing these issues necessitates improving isolation techniques to enhance purity, simplifying workflows to increase efficiency, and developing more sensitive and robust detection platforms. Additionally, the high cost of rapid and high-purity isolation methods remains a barrier to large-scale applications [111]. Although most studies are in vitro, further development of animal models and human trials is needed to ensure safety, specificity, and efficacy for clinical translation [111,112,113]. Thus, developing advanced tools for characterizing exosomal signatures and isolating tumor-specific exosomes is crucial to realizing the full potential of exosome-based applications.

In conclusion, while exosomes hold great promise as cancer biomarkers, significant technical challenges remain in their isolation, purification, and detection. Future research must focus on technological advancements and standardization processes to fully explore the potential of exosomes in cancer diagnostics.

## 3. Methodological Advances in Nanopore-Based Exosome Analysis

In recent years, biosensors have gained widespread attention from scientists and technologists worldwide for their potential to achieve higher sensitivity in exosome detection and more efficient separation [35,36,37]. Compared to traditional methods, biosensors offer significant advantages such as low cost, ease of operation, rapid response, high sensitivity, excellent specificity, and multiplexing capabilities, showcasing their vast application potential [38]. Nanopore sensors are broadly categorized into two main types based on their chemical composition: biological and solid-state nanopores [114]. Biological nanopores are formed through the spontaneous assembly of reproducible amino acid-based biopolymers within a planar lipid bilayer (PLB) [115]. The most extensively studied biological nanopore is α-hemolysin (αHL), a toxic heptameric protein secreted as a monomer by *Staphylococcus aureus* [116]. Fully assembled αHL nanopores exhibit excellent signal reproducibility across experiments. In 1996, John J. Kasianowicz demonstrated for the first time that a single DNA molecule could pass through the αHL protein nanopore (with a narrowest point of approximately 1.4 nm) and produce characteristic current changes, establishing the experimental basis for subsequent nanopore sequencing research [117]. In contrast to biological nanopores, solid-state nanopores are made from inorganic materials such as graphene, carbon nanotubes [118,119,120], glass [121], silicon, PDMS, and polycarbonate. These nanopores are fabricated by milling nanoscale holes into membranes of solid-state materials using high-powered electron beams or chemical etching techniques. Solid-state nanopores offer greater material diversity and functionality compared to biological nanopores [116]. Nanopore devices integrate efficient exosome separation with precise detection, enabling automated guidance, enrichment, and analysis of exosomes. This synergy opens new technical pathways for exosome research and significantly enhances the application of biosensors in processing complex biological samples. In the following sections, we will explore various nanopore-based methods for exosome detection and isolation, along with their potential applications in cancer diagnostics.

### 3.1. High-Throughput Exosome Separation Driven by Magnetic Nanopore Technology

**Figure 1:**
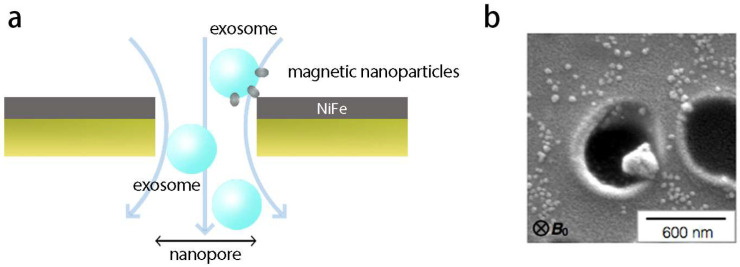
Exosome capture based on ExoTENPO. (**a**) High-throughput exosome separation using magnetic nanopores. (**b**) SEM image displaying exosomes captured at the edges of ExoTENPO pores. The small spherical objects are unbound magnetic nanoparticles (MNPs) [39]. Copyright 2017 ACS Nano.

Due to the nanoscale size of exosomes, precise detection with conventional microfluidic techniques presents challenges. Moreover, scaling up microfluidic technologies for exosome processing often leads to decreased device throughput. To address this, Ko et al. developed a track-etched magnetic nanopore (TENPO) sorting technology for capturing exosomes from plasma. By using a large-scale, parallelized nanopore immunomagnetic trapping system, they achieved high-precision exosome sorting at high flow rates (Figure 1a) [39,122,123,124].

Researchers fabricated magnetic nanopore chips by thermally evaporating a 200 nm soft magnetic NiFe layer and a 30 nm Au passivation layer onto track-etched polycarbonate membranes. These chips contain millions of nano-fluidic exosome isolation units [39,124]. In practical application, exosomes are first tagged with biotinylated antibodies and then attached to anti-biotin magnetic nanoparticles (MNPs). When samples flow through the chip at high flow rates (φ > 10 mL/h), exosomes labeled with magnetic tags are captured by the magnetic nanopores under the influence of magnetic electrophoresis, while unlabeled particles are washed out with the fluid (Figure 1b) [39]. Moreover, the Exosome Track-Etched Magnetic Nanopore (ExoTENPO) chip features a novel design that rotates traditional nano-fluidic separation by 90 degrees. This design shifts the magnetic traps from the interior of the channels to the pore edges, preventing clogging and enabling robust processing of raw samples, such as untreated serum or plasma [39].

Although TENPO technology has made significant progress in exosome isolation, challenges such as exosome heterogeneity and interference from background particles remain as major obstacles for separation techniques. To solve these issues, Lin et al. optimized TENPO technology by using an immunomagnetic sorting method targeting surface proteins. Biotinylated antibodies were used to label exosome surface markers, which were conjugated with streptavidin-coated MNPs. EVs with high expression of specific surface markers are strongly labeled, while those with low or no expression are weakly labeled. Furthermore, Lin et al. balanced recovery efficiency and nonspecific capture by fine-tuning parameters such as pore size, flow rate, and the number of tandem membrane units [39,125].

In summary, compared with traditional EV isolation methods, TENPO not only enhances processing capacity and sample throughput via parallelized immunomagnetic separation but also isolates EV subpopulations from complex media. By using etching and vapor deposition techniques to create magnetic nanopores, TENPO avoids costly, less scalable photolithographic processes, making it more economical and scalable for clinical applications [39,125].

### 3.2. Application of Electrochemical Signal Techniques in Precise Exosome Detection

In recent years, nanoelectrodes and nanopipettes have attracted significant attention for their applications in detecting analytes at the single-cell and subcellular levels [46,126,127]. These tools can be precisely placed within individual cells, with step resolutions of less than 100 nanometers. With advancements in micromanipulation technology, the resolution has been further enhanced, enabling subcellular analyte detection [126,127]. Electrochemical measurements with high temporal resolution can record single-molecule or particulate events. Thus, nanoelectrodes and nanopipettes are powerful electrochemical tools with high spatial and temporal resolution.

**Figure 2:**
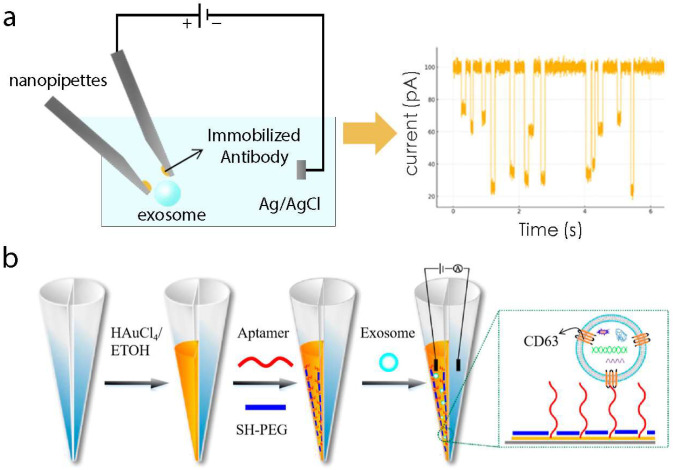
Exosome detection based on electrochemical resistive pulse (ERP) Technology. (**a**) Schematic diagram of the ERP experiment for exosomes through carbon nanopipettes (CNPs). (**b**) Schematic of the assembly process of a DNA-functionalized dual nanopore biosensor for exosome detection [46]. Copyright 2023 ACS Sens.

Electrochemical resistive pulse (ERP) sensing is an innovative technique using nanopipettes, designed to measure individual entities [128,129,130,131]. Jia et al. employed this method to detect single extracellular EVs released by specific cells [44]. Using carbon nanopipettes (CNPs) as electrodes, they measured resistive pulses to identify exosomes and analyzed their internal redox species using vesicle amperometry (Figure 2a) [132,133,134]. When redox-active species within the vesicles collide with the CNPs’ inner wall, oxidation reactions are triggered, resulting in a current spike after the resistive pulse.

In addition to CNPs, various electrochemical techniques based on glass nanopipettes have been developed for studying target analytes. Feng et al. developed a multifunctional nanoelectrode–nanopore nanopipettes (MNNPs) to detect exosomes from HeLa cells [45]. The MNNPs integrates two sensing units at the nanopipette tip: a nanopore and a carbon nanoelectrode (CNE). Functionalizing the CNE surface with anti-CD63 antibodies allows the MNNPs to specifically recognize exosomes. Experiments showed that anti-CD63-modified MNNPs detected exosomes precisely, while unmodified MNNPs had limited performance. This enhancement is likely due to the anti-CD63 modification, which enriched exosomes at the device tip and minimized interference from non-exosomal particles. However, this study highlighted a limitation in the detection lifespan of anti-CD63-modified MNNPs, suggesting the need for further research to improve their stability and reusability. Despite this limitation, the MNNPs combine well-defined surface chemistry on both the CNE and nanopore with the benefits of micromanipulation techniques and nanoscale electrode dimensions, enabling detection and analysis of live-cell secreted exosomes at the single-entity level. Glass nanopipettes are simple to fabricate and operate, and their cost-effectiveness makes MNNPs a promising tool for biomedical research and clinical diagnostics [131].

Recently, dual-nanopore biosensors with a dual nanopipette structure have been used for single-molecule and single-cell analyses due to their unique design [135]. Zhang et al. developed a DNA aptamer-based dual-nanopore biosensor by integrating ERP sensing with dual-nanopore technology (Figure 2b) [46]. In this system, thiol-modified DNA aptamers immobilized on gold-coated nanopores capture CD63 proteins on the exosome surface, reducing the effective pore size. This asymmetric structure allows exosome binding to induce a reduction in ionic current, enabling exosome concentration quantification. Compared with single-nanopore designs, dual-nanopore biosensors form an internal circuit that eliminates cell membrane interference and minimizes noise from membrane signals. While other EVs express CD63 on their surface, the 100 nm tip size of the dual nanopores selectively allows exosomes to pass through, excluding larger EVs. This is a unique advantage of nanopore technology in exosome detection.

### 3.3. Innovative Applications of Nanomaterial Assembly in Exosome Enrichment and Detection

**Figure 3:**
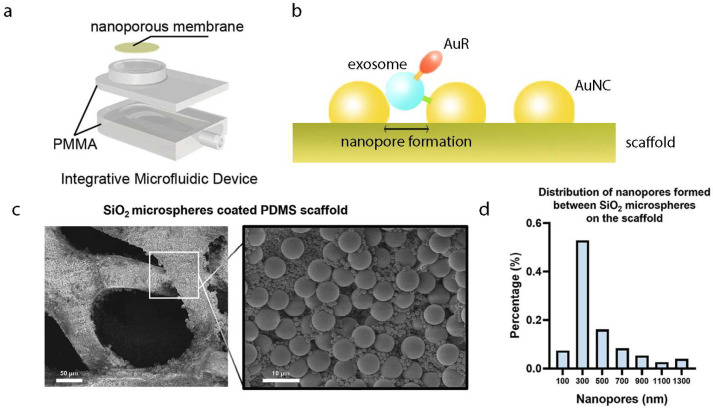
Nanomaterials assembly device for exosome separation and detection. (**a**) Design of an integrated microfluidic device with AuNC membrane [42]. Copyright 2020 Biosens Bioelectron. (**b**) Schematic diagram of AuNC–exosome–AuR structure for exosome detection. (**c**) SEM images and schematic diagram of a PDMS scaffold coated with SiO_2_ microspheres (denoted as SiO_2_/PDMS scaffold). The magnified view on the right shows detailed features [43]. Copyright 2024Adv Sci (Weinh). (**d**) Distribution of nanopores formed between the SiO_2_ microspheres deposited on the scaffold [43]. Copyright 2024Adv Sci (Weinh).

Yang et al. developed a novel microfluidic device (Figure 3a), which features polymethyl methacrylate (PMMA) base and a nanoporous gold nanocluster (AuNC) membrane. The AuNC membrane is fabricated by depositing cone-shaped gold nanoparticles on an anodized aluminum oxide (AAO) template, forming a membrane with adjustable pore sizes, fine-tuned by varying the gold layer thickness through ion sputtering. The membrane surface is functionalized with a primary antibody (capture antibody) to bind exosomes in urine. Next, gold nanorod (AuR) probes functionalized with a secondary antibody (detection antibody) are added, forming an AuNC–exosome–AuR sandwich structure (Figure 3b) [42]. The complex can be visualized under a dark-field microscope due to resonance Rayleigh scattering, enabling exosome detection [136].

To identify more effective cancer biomarkers, researchers have increasingly focused on exosomes, which show significant potential in tumor screening and liquid biopsy. To address inefficiencies in current exosome enrichment techniques, Yi et al. developed a 3D hierarchical porous chip coated with silicon dioxide (SiO_2_) microspheres [43]. The chip was fabricated using a sacrificial template method to create a continuous porous PDMS scaffold. This scaffold was modified with 1–5 µm carboxylated silica microspheres, resulting in nanopores smaller than 500 nm. Finally, the scaffold was functionalized with streptavidin (SA) and biotinylated anti-CD63 antibodies (Figure 3c,d). The 3D porous structure of the SiO_2_ scaffold promotes the transition from laminar to turbulent fluid flow, increasing the frequency of exosome collisions with the substrate. Simultaneously, the nanoporous SiO_2_ microspheres increase surface area, reduce boundary effects, and enhance exosome binding to the scaffold, improving capture efficiency. Notably, this method enabled direct exosome enrichment from plasma with a detection limit as low as 10,000 particles/mL. The analysis required only 40 µL of plasma and was completed in 10 min. Although some pores formed by SiO_2_ microspheres may not strictly be considered nanopores, this study emphasizes that smaller pore sizes are more effective in capturing exosomes. Therefore, further reducing pore size is a promising avenue for advancing this technology.

### 3.4. Enhancing Exosome Detection Sensitivity Using Plasmon Resonance

The physical properties of nanomaterials are intrinsically linked to their size, shape, and composition. By incorporating nanostructures on noble metal substrates, innovative detection and analysis methods have been developed. For instance, plasmonic nanomaterials interact uniquely with light across various wavelengths [136,137,138,139].

Im et al. developed a detection method based on nanoplasmonic exosome technology (nPLEX), which uses periodic nanopore arrays for surface plasmon resonance [40]. The nPLEX sensor features a gold nanopore array with a diameter of 200 nm and a periodicity of 450 nm. By adjusting the periodicity of the nanopores, the electromagnetic field range overlaps with the size of the exosomes, thereby maximizing detection sensitivity. Exosome binding is detected by monitoring local refractive index changes, which can be quantitatively measured by either the wavelength shift or the intensity changes at a fixed wavelength. Studies have demonstrated that the detection limit of nPLEX is approximately 670 aM, with sensitivity 104-fold higher than Western blot analysis and 102-fold higher than chemiluminescence ELISA. Additionally, the platform amplifies signals through secondary labeling, improving sensitivity, accelerating detection, and reducing sample requirements.

**Figure 4:**
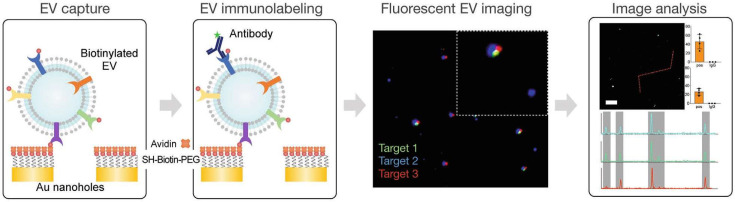
Precise analysis of exosomes enabled by nPLEX technology. nPLEX-FL technology for single EVs analysis. EVs are captured on the nanopore surface, immunostained with fluorescent probes, and imaged across fluorescence channels for intensity analysis [41]. Copyright 2020 Adv Biosyst.

To enhance sensitivity, Min et al. developed a fluorescence-enhanced version of nPLEX technology (nPLEX-FL), which amplifies exosome fluorescence signals via periodic gold nanopores inducing surface plasmon resonance (Figure 4) [41]. Fluorescence-based detection methods are widely employed for identifying various biological targets [140,141,142,143]. However, the limited surface area and volume of exosomes restrict the abundance of biomarkers, limiting the fluorescence signal generated by individual exosomes [144,145]. nPLEX-FL technology amplifies fluorescence signals by an order of magnitude [41]. In this system, biotinylated EVs are captured on a biotinylated PEG-modified gold nanopore surface, followed by immunostaining with fluorescently labeled antibodies. Fluorescence microscopy is used to image and quantify the fluorescence intensity and quantity of the exosomes (Figure 4).

Based on plasmonic resonance principles, Yang et al. enhanced the sensitivity of nanopore-based exosome detection and isolation [42,146,147,148]. In the AuNC–exosome–AuR complex, 50 nm gold nanocones (AuNCs) scatter green light, while 40–80 nm gold nanorods (AuRs) scatter red light. When the distance between AuNCs and AuRs is less than 200 nm, plasmonic coupling shifts the scattered light spectrum to the yellow region and boosts scattering intensity. This resonant Rayleigh scattering effect shifts the wavelength of the scattering spectrum of the AuR-based complex, while the plasmonic effect significantly amplifies the scattering intensity. This approach enables ultrasensitive detection and quantification of exosomes under dark-field microscopy, with a detection limit of 1000 particles/mL [42].

## 4. Diverse Applications of Nanopore Technologies in Exosome Separation and Detection for Cancer Screening Across Tumor Types

To improve the diagnosis of pancreatic ductal PDAC and enable earlier, more treatable detection, Ko et al. developed a method to detect exosomal miRNA biomarkers using the TENPO device. By integrating machine learning algorithms, this method distinguishes miRNA biomarkers among healthy mice, pancreatic intraepithelial neoplasia (PanIN), and PDAC, achieving diagnostic accuracy of up to 88%. These findings provide strong technical support for screening high-risk individuals for pancreatic cancer [122]. With its efficiency, cost-effectiveness, and high throughput, TENPO shows significant potential in accelerating exosome analysis from patient blood samples. It is well-suited for processing diverse, complex clinical specimens, enabling early detection of cancer biomarkers, advancing diagnosis and personalized treatment, and driving progress in cancer diagnostics.

Studies by Jia et al. have shown that the ERP technique distinguishes ROS/RNS in EVs from non-transformed and metastatic human breast cells, and further differentiates various breast cancer cell lines at the single-cell level [44]. Compared to intracellular amperometry [149], the ERP technique is easier to operate and faster, making it an ideal platform for investigating ROS/RNS-related oncogenic mechanisms. It also shows potential for the early diagnosis of aggressive triple-negative breast cancer. Moreover, this technique allows for real-time monitoring of single cells under induced conditions, enabling the evaluation of therapies targeting breast cancer cells by inducing oxidative stress [44].

The AuNC–exosome–AuR microfluidic device developed by Yang et al. can isolate and detect lung cancer-specific exosomes from patient urine [42]. LRG1 is significantly expressed in exosomes from the urine of lung cancer patients. Clinical analysis of urine samples showed stronger anti-LRG1-AuR signals in lung cancer patients than in healthy controls, suggesting the approach’s potential for identifying early-stage lung cancer patients. Furthermore, the detection system is highly sensitive, easy to operate, and promises to accelerate the clinical translation of exosome-based cancer diagnostics. Using the 3D hierarchical porous chip method developed by Yi et al. [43], two exosomal lncRNA biomarkers (*LUCAT-1* and *EGFR-AS-1*) for HCC diagnosis were identified. The levels of these biomarkers increased progressively from stage I to stage IV of HCC. Combined with clinical markers like AFP and DCP, this method improved diagnostic accuracy, particularly in distinguishing high-risk lesions from early-stage HCC during recurrence detection. This approach provides a valuable tool for personalized HCC screening, enhancing early detection and prognosis.

By applying the nPLEX technology developed by Im et al., exosomes derived from ovarian cancer cells were identified in ascitic samples from ovarian cancer patients, based on biomarkers such as CD24 and EpCAM. This method shows promise in distinguishing ovarian cancer patients from healthy controls, particularly in monitoring therapeutic response [40]. The nPLEX-FL technology developed by Min et al. provides a powerful signal amplification strategy for exosome detection, enhancing sensitivity and enabling multiplex biomarker analysis at the single EV level. In experiments with glioblastoma cell lines Gli 36-WT and Gli 36-EGFRvIII, nPLEX-FL showed strong performance in detecting cancer cell-derived exosomes in human plasma, comparable to standard quantitative methods such as Western blotting and droplet digital PCR [41]. Overall, the nPLEX-FL technology holds significant diagnostic potential, particularly in the detection and analysis of cancer-derived exosomes, making it a promising tool for clinical applications.

The clinical translation of nanopore-based exosome technologies has the potential to revolutionize patient care by enhancing early detection, personalizing treatment, and enabling non-invasive monitoring. By enabling ultrasensitive detection of tumor-derived exosomes, nanopore technology could identify malignancies at precancerous or localized stages, thereby enabling effective intervention.

## 5. Comparative Analysis of Nanopore-Based Approaches for Exosome Detection in Tumor Screening

The diverse nanopore technologies discussed in this review each have unique strengths and limitations in exosome detection and isolation. A critical comparison of these methods is essential to guide their optimal application in both clinical and research settings. In the following analysis, we evaluate four key nanopore-based approaches: magnetic nanopores, electrochemical sensing, nanomaterial assemblies, and plasmon resonance. These approaches are compared across several parameters, including sensitivity, specificity, throughput, operational complexity, clinical application, and cost-effectiveness (Table 1).

Magnetic nanopore technology excels in high-throughput processing (>10 mL/h), enabling rapid isolation of exosomes directly from raw biological samples like untreated plasma, making it indispensable for large-scale screening applications such as pancreatic cancer miRNA profiling. However, its moderate sensitivity and potential nonspecific binding of magnetic nanoparticles limit its resolution for heterogeneous exosome subpopulations [39,122,123,124]. Electrochemical sensing, characterized by single-molecule resolution and real-time dynamic analysis, provides unparalleled insights into redox activity within exosomes, such as ROS/RNS dynamics in metastatic breast cancer. Despite its high sensitivity, low throughput and susceptibility to environmental noise restrict its use to specialized mechanistic studies rather than routine clinical workflows [44,45,46]. Nanomaterial assembly stands out for its ultrahigh sensitivity (detection limit ~1000 particles/mL) and rapid workflow (10 min), particularly effective in early-stage cancer detection using biofluids like urine (e.g., lung cancer via LRG1 biomarkers). Yet, its operational complexity and reliance on precise nanomaterial synthesis hinder scalability for large-scale applications [42,43]. In contrast, plasmon resonance leverages surface-enhanced techniques like nPLEX-FL to achieve ultrasensitive multiplex detection, amplifying fluorescence signals tenfold for precise molecular subtyping (e.g., ovarian cancer ascites analysis). While its ability to profile multiple biomarkers simultaneously is groundbreaking, high equipment costs and demanding optical alignment requirements confine its use to specialized research or high-end diagnostics [40,41,42].

Collectively, magnetic nanopores dominate clinical screening due to their scalability, while electrochemical and plasmonic methods cater to precision research. Nanomaterial-based approaches bridge sensitivity and speed but face cost and complexity barriers. Future advancements must prioritize integration of these technologies—combining high throughput with multiplex precision—to unlock their full potential in liquid biopsy and personalized oncology.

## 6. Commercial Products for Cancer Diagnosis Based on Exosomes

In recent years, significant progress has been made in the development of exosome-based diagnostic products, with nanopore technology playing an increasingly important role. These commercial products provide a glimpse into the revolutionary potential of nanopore-based exosome detection in cancer diagnostics, but their clinical translation and real-world application still face challenges.

One notable example is Exosome Diagnostics’ ExoDx Prostate (IntelliScore), the world’s first non-invasive prostate cancer diagnostic product based on urinary exosomal RNA biomarkers [150,151]. By analyzing the expression profiles of RNA biomarkers such as *ERG*, *PCA3*, and *SPDEF* in exosomes, this technology can effectively distinguish high-risk prostate cancer (Gleason score ≥ 7) from low-risk cases. Validation studies have shown a sensitivity of 92% and specificity of 34%, reducing unnecessary biopsies by 27% [68,150]. This product has been incorporated into the National Comprehensive Cancer Network (NCCN) guidelines for prostate cancer detection, providing support for over 50,000 patients [68,150,151]. Similarly, Exosome Diagnostics has expanded its product range to include tests for lung cancer and other malignancies, further solidifying its role in liquid biopsy applications.

Another important development is the Exoid system, developed by Izon Science, which integrates nanopore technology to analyze exosomes and nanoparticles. The system provides high-resolution, single-particle detection, enabling precise measurements of exosome size [152,153], concentration [154,155], and surface charge [156,157]. This product is particularly useful for understanding exosome populations in complex biological fluids and is applicable to cancer diagnostics and biomarker discovery. Despite its potential, this technology remains largely confined to laboratory research, with challenges to widespread adoption in routine clinical practice.

Furthermore, Excipio Technologies has developed a flexible platform for exosome isolation using Vn96 peptides that specifically bind to heat shock proteins on the surface of exosomes, accelerating their isolation and improving diagnostic sensitivity [158]. This technology offers an effective alternative to traditional isolation methods, such as ultracentrifugation, significantly reducing processing time while maintaining high purity and specificity. However, these products still face challenges in terms of scalability and cost-effectiveness for clinical applications.

In conclusion, while commercial products leveraging nanopore technology for exosome detection show great promise, further optimization, clinical validation, and integration into standard diagnostic workflows are needed. Overcoming these hurdles will unlock the full potential of nanopore-based exosome technologies in clinical settings, particularly for early cancer detection, personalized treatment, and ongoing patient monitoring.

## 7. Discussion and Future Perspectives

Nanopore technology has shown significant potential for tumor screening, especially in early cancer detection through exosome-based liquid biopsy. However, it remains largely confined to laboratory research. To fully translate this technology into clinical applications, several key research areas and challenges must be addressed.

### 7.1. Improving Sensitivity and Specificity

While nanopore technology is already capable of single-molecule detection, further enhancing the sensitivity to detect low-abundance exosomes and biomarkers is crucial. Future research should focus on refining nanopore designs, such as reducing pore sizes and optimizing membrane properties, to increase the resolution and detection speed. Moreover, functionalizing nanopores with specific ligands or antibodies can help achieve higher selectivity and stability, making it easier to isolate cancer-specific exosomes amidst complex biological samples.

### 7.2. Integration with Machine Learning and Data Analysis

Optimizing machine learning algorithms is crucial for advancing nanopore-based exosome detection and isolation. These algorithms can identify and analyze features in electrical signal data, significantly improving detection accuracy and efficiency [159,160]. While electrical signals are widely used for exosome detection, machine learning has not been fully leveraged for in-depth signal analysis [44,45]. Future research should focus on utilizing machine learning to enhance signal resolution, improve the signal-to-noise ratio, reduce noise interference, and refine the sensitivity and specificity of electrical signals, making them more adaptable to complex biological environments. Machine learning builds models to analyze known data and predict unknown outcomes. Algorithms such as Support Vector Machines (SVMs), Random Forest (RF), and Neural Networks (NNs) are commonly used to classify and predict complex data from multiple signal sources [161,162]. For instance, Wu et al. used K-Nearest Neighbors (KNNs) and SVMs to analyze fluorescent signals from urine-derived exosomes, successfully classifying multiple diseases [163]. However, the application of machine learning is often limited by dataset size and quality, with limited clinical samples potentially affecting model accuracy. Future research should focus on expanding datasets and developing more robust algorithms to improve model reliability and practicality [162]. Overall, continual optimization of machine learning algorithms, particularly in handling high-dimensional data and complex biomolecule recognition, will enhance signal analysis and promote their application in clinical diagnostics.

### 7.3. Broadening Applications Beyond Oncology

While nanopore technology has primarily focused on cancer diagnostics, its potential applications extend well beyond oncology. Studies have demonstrated its promise in the early detection of viral infections [164], as well as in diagnosing and monitoring neurodegenerative diseases [165] and cardiovascular conditions [166,167]. In hematologic malignancies, such as acute leukemia, nanopore technologies could facilitate the detection of tumor-derived exosomes in blood or other biofluids, enabling more rapid diagnosis compared to traditional methods. This approach can potentially reduce the need for invasive procedures like bone marrow biopsies, offering a quicker, safer, and more cost-effective way to monitor disease progression and therapeutic response. Furthermore, in chronic lymphocytic leukemia (CLL), exosomal *miR-223* is downregulated, highlighting the diagnostic value of miRNAs in liquid biopsies for blood cancers [168]. Expanding research to include these areas could significantly broaden the impact of nanopore technology in precision medicine. However, the successful integration of nanopore technology into these diverse fields presents several challenges, such as optimizing nanopore systems for different biomarkers across diseases, standardizing protocols, and ensuring the technology’s robustness across patient populations. Addressing these challenges will be key to unlocking the full potential of nanopore technology in both oncology and beyond.

### 7.4. Multiplexing and Multi-Biomarker Detection

One key advantage of nanopore technology is its multiplexing capability, which can be developed into parallel detection platforms for analyzing multiple biomarkers simultaneously. This improves detection efficiency and broadens the scope of a patient’s health assessment. The fluorescence-enhanced version of nPLEX-FL, developed by Min et al., highlights current multiplexing limitations, as fluorescence microscopy systems typically support only three to four colors. Future developments should focus on enhancing the multiplexing capabilities of nanopore systems, potentially through innovations like cyclic imaging or more advanced labeling techniques, to enable the simultaneous detection of multiple biomarkers for comprehensive cancer screening [41,169,170]. By enabling multiplexed detection and parallel processing, nanopore technology holds the potential to establish a robust biosensing network, advancing precision medicine.

### 7.5. Clinical Validation and Regulatory Challenges

Despite promising results in research settings, the clinical translation of nanopore-based technologies remains a significant hurdle. The lack of standardized protocols for exosome isolation, characterization, and analysis has led to inconsistencies across studies. Furthermore, nanopore devices must undergo rigorous clinical validation, including large-scale clinical trials and regulatory approval, before they can be widely implemented in healthcare settings. While some commercial products have emerged that apply nanopore technology in exosome research, their use is still limited, with very few reaching the stage of application in medical diagnostics or tumor screening. Future efforts must address these challenges by establishing clear standards, conducting comprehensive clinical studies to demonstrate diagnostic accuracy and reliability, and overcoming barriers to commercialization in the healthcare sector.

While nanopore-based biosensing technologies for exosome detection and separation are still in the early stages of clinical application, advances in cost control, data processing, integration, and device manufacturing are expected to gradually overcome existing challenges. As nanopore technology evolves, it is poised to play a critical role in future cancer diagnosis and monitoring, offering more efficient and precise solutions for clinical practice.

## Figures and Tables

**Table 1:** Comparative analysis of nanopore approaches.

Nanopore Approach	Magnetic Nanopores	Electrochemical Sensing	Nanomaterial Assembly	Plasmonic Nanopores
Core Function	High-throughput isolation	Single-particle detection	Ultrasensitive detection	Multiplex biomarker profiling
Sensitivity/ Detection Limit (LOD)	Moderate (Sensitivity improves with magnetic nanoparticles and functionalization)	High (Single-molecule detection)	High /~1000 particles/mL (Signal amplification with nanomaterials improves detection limits)	High /~670 aM (Detects low abundance biomarkers)
Specificity	High (Capable of effectively distinguishing target exosomes from other particles)	High (Selective recognition of target molecules after functionalization)	High (Functionalized nanomaterials enhance the recognition of target exosomes)	Very high (Utilizes resonance to detect molecular interactions)
Throughput	High	Moderate	Moderate	Low
Operational Complexity	Low (Automated systems)	High (Requires skilled handling)	Moderate (Nanomaterial synthesis)	High (Optical alignment)
Clinical Application	Used in exosome isolation and tumor biomarker detection, potential for liquid biopsy applications	Not widely adopted yet in clinical trials, but showing potential for real-time cancer detection	Limited clinical application; mainly in research or early stage commercial use	Not widely used in clinical settings yet, mostly in research and specialized diagnostics
Cost	Low to moderate (Magnetic nanoparticles and separation equipment)	Moderate (Depends on sensor integration and detection setup)	Moderate to high (High cost of nanomaterials and assembly processes)	High (Requires specialized equipment and maintenance)

## Abbreviations

AFP	Alpha-Fetoprotein
AuNC	Gold Nanocluster
AuR	Gold Nanorod
BPH	Benign Prostatic Hyperplasia
CA15-3	Cancer Antigen 15-3
CA19-9	Carbohydrate Antigen 19-9
CD63	Cluster of Differentiation 63 (Exosome surface markers)
CNPs	Carbon Nanopipettes
CRPC	Castration-Resistant Prostate Cancer
ctDNA	Circulating Tumor DNA
CTCs	Circulating Tumor Cells
DCP	Des-Gamma-Carboxy Prothrombin
EGFR	Epidermal Growth Factor Receptor
ERP	Electrochemical Resistive Pulse
EVs	Extracellular Vesicles
GPC-1	Glypican-1
HCC	Hepatocellular Carcinoma
ILVs	Intraluminal Vesicles
MNNPs	Multifunctional Nanoelectrode-Nanopore Nanopipettes
MVBs	Multivesicular Bodies
nPLEX	Nanoplasmonic Exosome Technology
NSCLC	Non-Small Cell Lung Cancer
NCCN	National Comprehensive Cancer Network
PDAC	Pancreatic Ductal Adenocarcinoma
PMMA	Polymethyl Methacrylate
PSA	Prostate-Specific Antigen
ROS/RNS	Reactive Oxygen Species/Reactive Nitrogen Species
SEM	Scanning Electron Microscope
TSP1	Thrombospondin-1
